# Parameter Estimation of Poisson–Gaussian Signal-Dependent Noise from Single Image of CMOS/CCD Image Sensor Using Local Binary Cyclic Jumping

**DOI:** 10.3390/s21248330

**Published:** 2021-12-13

**Authors:** Jinyu Li, Yuqian Wu, Yu Zhang, Jufeng Zhao, Yingsong Si

**Affiliations:** School of Electronics and Information, Hangzhou Dianzi University, Hangzhou 310018, China; lijinyu@eagle-ai.com (J.L.); wyq0807@hdu.edu.cn (Y.W.); dabaozjf@hdu.edu.cn (J.Z.); ysongsi@163.com (Y.S.)

**Keywords:** noise parameter estimation, Poisson–Gaussian noise model, weakly textured blocks image selection

## Abstract

Since signal-dependent noise in a local weak texture region of a noisy image is approximated as additive noise, the corresponding noise parameters can be estimated from a given set of weakly textured image blocks. As a result, the meticulous selection of weakly textured image blocks plays a decisive role to estimate the noise parameters accurately. The existing methods consider the finite directions of the texture of image blocks or directly use the average value of an image block to select the weakly textured image block, which can result in errors. To overcome the drawbacks of the existing methods, this paper proposes a novel noise parameter estimation method using local binary cyclic jumping to aid in the selection of these weakly textured image blocks. The texture intensity of the image block is first defined by the cumulative average of the LBCJ information in the eight neighborhoods around the pixel, and, subsequently, the threshold is set for selecting weakly textured image blocks through texture intensity distribution of the image blocks and inverse binomial cumulative function. The experimental results reveal that the proposed method outperforms the existing alternative algorithms by 23% and 22% for the evaluative measures of MSE (a) and MSE (b), respectively.

## 1. Introduction

With the rapid development of complementary metal oxide semiconductor (CMOS) technologies, CMOS image sensors have become popular with consumer and vehicle electronics, telemedicine, video surveillance, space exploration, fluorescence detection, and so on [1,2,3,4]. However, images generated by these sensors inevitably contain noise, owing to their internal structure, which results in image quality degradation [5,6], and thus, estimating these noise parameters accurately assumes paramount importance in improving the performance of denoising algorithms [3,4,7,8,9]. For CMOS image sensors, a signal-dependent noise model, such as the Poisson–Gaussian model, can more accurately delineate the noise characteristics than an additive channel-dependent noise model [10,11,12,13,14,15,16,17,18,19].

Past studies have engineered various noise parameter estimations methods to adopt the Poisson–Gaussian signal-dependent noise model for CMOS image sensors and achieved satisfactory results. One kind is based on deep learning, which depends on the ability of the convolutional neural network in memorizing training data [20,21,22,23,24,25]. The other is based on calculation from single image. The latter is broadly classified into two categories viz. methods based on variance stabilization transformation (VST) and methods based on fitting sample pairs.

## 2. Related Work

Parameter estimation methods based on VST transform signal-dependent noise to additive white Gaussian noise (AWGN) in the transform domain. Pyatykh and Hesser [12] devised a VST-based parameter estimation method using an optimization procedure, where the input noisy image was initially transformed into a corrupted AWGN and later analyzed and estimated using principal component analysis (PCA) in the transform domain. Mäkitalo and Foi [13] developed an algorithm for estimating noise in these image sensors by coalescing an iterative VST and resultant estimated noise of the AWGN. However, the premise of VST asserts that the variance of the image data be negligible, thereby, rendering these methods unsuitable for images with large noise variances. The VST-based noise level parameter estimation algorithm needs to use the real noise model parameters when performing the VST, and estimating the noise model parameters is exactly the purpose of the algorithm. This iterative relationship will lead to the unstable estimation effect of such methods at the actual running time.

Parameter estimation methods based on fitting sample pairs focus on accurately identifying weakly textured image blocks in a noisy image. This kind of estimation method can be divided into four categories according to the definition of the texture intensity of weakly textured image blocks, they are methods based on variance or standard deviation, methods based on gradients, methods based on image grey entropy, and methods based on histograms.

The method based on variance or standard deviation was performed by wavelet analysis [10,15,16]. It separates the noise in the image block into the high-frequency area, while retaining the image information containing the image texture in the low-frequency area, then calculate the standard deviation or variance of the low-frequency area of the image block, measure the texture intensity of the image block with the size of its standard deviation or variance, and set the threshold to select relatively flat image blocks. However, while separating the image noise, part of the texture and edge information also comes to the high frequency area, which leads to inaccurate texture intensity calculation. At the same time, the low frequency part of losing part of the texture information is less than the real value when calculated as the pixel intensity estimate, while the high frequency part containing the texture will be larger than the real noise variance estimate, which eventually leads to the noise variance estimate is larger at the same pixel intensity level. 

To this effect, an image gradient matrix [14,17] is harnessed to evaluate the image texture intensity. The authors compute the texture intensity of the image block as the sum of the eigenvalues of the image block gradient covariance matrix, which in turn contributes to the selection of weakly textured image blocks. However, the proposed method only considers the image pixel changes in the horizontal and vertical direction of the image block when calculating the texture strength, as opposed to all directions, and the resulting texture strength still varies from the real texture strength. 

In [18], a histogram of local mean values is employed to select image blocks with continuous pixel intensity as weakly textured image blocks. However, since image blocks with different texture intensities can still contain similar average pixel values, the selected weak texture image blocks may contain blocks with higher texture strength, thus making the estimated noise variance high, and this method may cause the pixel mean of the selected image blocks all concentrated in a certain range, so that the estimated noise level estimates of the noise variance in other pixel intensity regions can deviate greatly from the real values. 

In [19], the local grey entropy of the image blocks was used to select the weakly textured image blocks. The drawback of the method is that the grey entropy of the image is expressed as the bit average of the set of image gray scale levels, reflecting the average amount of information, but the number of pixels inside the image with different textures may also be the same, so the image grey entropy is not completely linearly correlated with its texture strength.

The key to the noise parameter estimation algorithm based on fitting sample pairs is the selection of weakly textured image blocks. However, the existing image block texture intensity definition methods cannot fully characterize the texture information of image blocks. To improve the overall accuracy of selecting these weakly textured image blocks and estimating the noise parameters of a noisy image thereafter, we propose a novel methodology based on local binary cyclic jumping as applied to a Poisson–Gaussian signal-dependent noise model. First, N×N image blocks are extracted from a noisy image. Next, the texture strength of the image blocks is defined and computed using local binary cyclic jumping. Then, the image blocks with weak texture strengths are nominated to elicit the set of sample pairs of pixel intensities and noise variances, and the traditional ordinary least squares is applied to fit the sample pairs, this iterative process will be terminated until the estimated noise variance remains unchanged and optimal noise parameters are obtained.

## 3. Methodology

### 3.1. Poisson–Gaussian Signal-Dependent Noise Model

The signal-dependent noise of a CMOS image sensor can be modelled as:

(1) $$I\left(i,j\right)=x\left(i,j\right)+\eta \left(i,j\right)=x\left(i,j\right)+\eta_{P}\left(x\left(i,j\right)\right)+\eta_{N}\left(i,j\right)$$

where i and j represent the row and column of a pixel in the image, respectively; I(i,j) denotes a pixel value of the noisy image at (i,j); η(i,j) denotes a value of the noise at (i,j); and x(i,j) denotes a pixel value of the noise-free image at (i,j). $\eta_{p}(x(i,j))$ represents the signal-dependent Poisson noise component of the photon noise resulting from fluctuations in the number of detected photons. 

Pixel values affected by the Poisson noise component pursue a Poisson distribution, whose variance and mean values are given by $\frac{1}{a}(x(i,j))$, as shown below:

(2) $$\frac{1}{a}(x(i,j)+\eta_{P}(x(i,j)))\sim P(\frac{1}{a}x(i,j))$$

where a is the photon noise parameter as per the quantum efficiency of the sensor. A large value of a is indicative of higher number of photons required to elicit a response from the sensor, thereby proportionally magnifying the impact of the photon noise on the image.

Furthermore, $\eta_{N}(i,j)$ is the signal-independent zero-mean Gaussian noise component that characterizes the thermal and electrical noise contributed by circuits:

(3) $$\eta_{N}(i,j)\sim N(0,b)$$

where b is the variance of the Gaussian noise. A large value of b is indicative of greater impact from thermal and electrical noise on the image. 

Since the mean and variance of the Poisson distribution are equal, it can be obtained by formula (2):

(4) $$E\left\{\frac{1}{a}\left(x\left(i,j\right)+\eta_{P}\left(x\left(i,j\right)\right)\right)\right\}=var\left\{\frac{1}{a}\left(x\left(i,j\right)+\eta_{P}\left(x\left(i,j\right)\right)\right)\right\}$$

Also because $E\left\{\frac{1}{a}\left(x\left(i,j\right)+\eta_{P}\left(x\left(i,j\right)\right)\right)\right\}=\frac{1}{a}x\left(i,j\right)+\frac{1}{a}E\left\{\eta_{P}\left(x\left(i,j\right)\right)\right\}$ and $E\left\{\frac{1}{a}\left(x\left(i,j\right)+\eta_{P}\left(x\left(i,j\right)\right)\right)\right\}=\frac{1}{a^{2}}var\left\{\eta_{P}\left(x\left(i,j\right)\right)\right\}=\frac{1}{a}x\left(i,j\right)$, it can be deduced that:

(5) $$E\left\{\eta_{P}\left(x\left(i,j\right)\right)\right\}=0\;var\left\{\eta_{P}\left(x\left(i,j\right)\right)\right\}=ax\left(i,j\right)$$

Therefore, the overall noise variance $\sigma^{2}(i,j)$ of a pixel at location (i,j) then can be given as:

(6) $$\sigma^{2}(i,j)=ax(i,j)+b$$

The goal of the proposed noise parameter estimation model is to obtain the values of a and b from a noisy image generated by the CMOS image sensor. 

### 3.2. Proposed Noise Parameter Estimation Model

The proposed algorithm comprises five principal modules, which are extraction of image blocks, defining and estimating texture strength using LBCJ, selection of weakly textured image blocks based on texture strength, estimating pixel intensity and variance sample pairs, and fitting sample pairs to elicit noise parameters.

①: Extraction of Image Blocks

The first module of the algorithm is to extract a certain number of image blocks with the same size from the noise image, which is to prepare for the later selection of weak texture image blocks as sample data. For a noisy image of size R×S, N×N-sized image blocks are extracted by sequentially making the central pixel traverse every pixel from the left to right column and top to bottom row. The size of N will have a certain impact on the accuracy of the estimation, and it will also affect the running time of the algorithm. If N is too small, it will increase the complexity and running time of the algorithm; if N is too large, it will affect the accuracy of the algorithm. According to [19], we set the size parameter N of the image block to 15 pixels in this research, to ensure a good estimation effect while taking into account faster algorithm execution efficiency. Then B_n=(R-N+1)×(S-N+1) image blocks were extracted from the supplied noisy image, where B_n is the total number of image blocks.

②: Defining and Estimating Texture Strength using Local Binary Cyclic Jumping

After extracting a certain number of image blocks from the noise image, these image blocks need to be selected, and some relatively flat image blocks with weak texture information are selected as image sample data for subsequent estimation. At this time, a judgment is required to measure the intensity of the image block texture. Therefore, a method based on local binary cyclic jumping is designed to define the texture intensity of image blocks.

**Figure 1:**
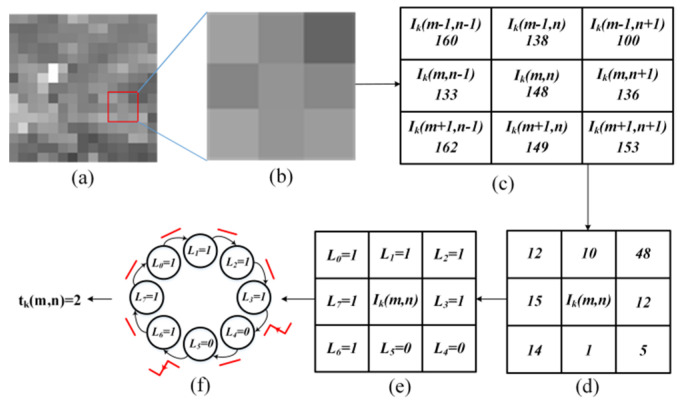
Principle of local binary cyclic jumping of central pixel $I_{k}(m,n)$. (**a**) *N* × *N*-sized image block $I_{k}$; (**b**) central point $I_{k}(m,n)$ and adjacent pixels in eight-neighbor connected domain; (**c**) pixel values of $I_{k}(m,n)$ and adjacent pixels in eight-neighbor connected domain; (**d**) absolute difference between central pixel and those in eight-neighbor connected domain; (**e**) binary values of corresponding pixels in eight-neighbor connected domain; (**f**) calculating the number of cyclic jumps of central pixel.

For an image block $I_{k}\left(k\in \left[1,B\_n\right]\right)$ of size *N* × *N*, as shown in Figure 1a, where m and n denote the row and column of a pixel, respectively, the central pixel is represented by $I_{k}(m,n)$ for m∈[2,N-1],n∈[2,N-1], and its eight-neighbor connected domain is shown in Figure 1b. The individual pixel values in eight-neighbor connected domain are shown in Figure 1c. Their corresponding binary forms are labelled $L_{0}$–$L_{7}$, as shown in Figure 1e.

Next, a local texture binary cyclic jumping is performed. For this, the absolute difference between the central pixel and every adjacent pixel is computed, as shown in Figure 1c,d. If this difference is greater than the predefined threshold value, the binary value of the corresponding pixel is set to 1, else 0, as shown in Figure 1d,e. The pixel intensities in the homogeneous region are close and according to [16,17], the maximal intensity difference of two pixels in the homogeneous region is set to 15. Since the absolute value is taken, the threshold is set to half of the maximum difference, which is 7.5. The binary value of the corresponding pixel can then be written as:

(7) $$L_{d}=\left\{ \begin{array}{l} 1,if\left|I_{k}\left(m,n\right)-I_{k}\left(m+u,n+v\right)\right|>\delta \\ 0,if\left|I_{k}\left(m,n\right)-I_{k}\left(m+u,n+v\right)\right|\leq \delta \end{array}\right.,d\in [0,7],u\in \left\{-1,0,1\right\},v\in \left\{-1,0,1\right\}$$

Likewise, after computing the binary values of pixels in the eight-neighbor connected domain, a local binary cyclic jumping is formed by arranging $L_{0}$–$L_{7}$ in a circular loop, as shown in Figure 1f. Then the cycle jumping number of the eight-bit binary circle sequence is calculated for the central pixel $I_{k}(m,n)$ by (8), which reflects texture intensity information of the central pixel:

(8) $$t_{k}(m,n)=J\_n\{L_{0}\sim L_{7}\}$$

where $J\_n\left\{L_{0}\sim L_{7}\right\}$ represents the number of cyclic transitions of binary values in the binary sequence composed of $L_{0}\sim L_{7}$, since it is a cyclic transitions, the number of transitions is independent of starting position.

The texture strength, $T_{k}$, of the image block $I_{k}$ is defined as average of the texture intensity information $t_{k}(m,n)$ around all central pixels $I_{k}(m,n)$ for m∈[2,N−1],n∈[2,N−1] in $I_{k}$:

(9) $$T_{k}=\frac{1}{{\left(N-2\right)}^{2}}\sum_{m=2}^{N-1}\sum_{n=2}^{N-1}t_{k}(m,n)$$

③: Selection of Weakly Textured Image Blocks Based on Texture Strength

After calculating the texture intensity of each image block, the weak texture image block needs to be selected according to the texture intensity. The key is to determine the threshold of the weakly textured image blocks. Therefore, a method based on texture intensity distribution of image blocks is designed to calculate the threshold of the weakly textured image blocks.

First, we derive the statistical distribution of texture intensity of a flat image block affected by noise: assume that the image block is a flat area before being contaminated by signal dependent noise, according to literature [18] and (1), for large photon counts, in a noisy flat patch, $I_{k}$ can be approximated as:

(10) $$I_{k}\left(m,n\right)=x_{k}\left(m,n\right)+\eta_{k}\left(m,n\right)$$

where $x_{k}(m,n)$ is the noise-free image data, $\eta_{k}\left(m,n\right)$ is the independent zero-mean Gaussian with the standard deviation σ(f), $\sigma^{2}\left(f\right)$ is the noise variance, and *f* is the intensity of the flat patch, The following deduction can be then acquired:

(11) $$\eta_{k}(m,n)\text{\textasciitilde{}}N(0,\sigma^{2}(f))$$

In the same way, the noise $\eta_{k}(m+u,n+v)$ in the image block $I_{k}$ which is adjacent to $I_{k}\left(m,n\right)$ also obeys the Gaussian distribution, with a mean value of 0 and a variance of $\sigma^{2}\left(f\right)$, as shown in the formula (12).

(12) $$\eta_{k}\left(m+u,n+v\right)\sim N\left(0,\sigma^{2}\left(f\right)\right),u\in \left\{-1,0,1\right\},v\in \left\{-1,0,1\right\}$$

As $I_{k}$ is a noisy flat patch, the $x_{k}$ on it is approximately same, and (13) is, hence, obtained.

(13) $$\begin{array}{l} I_{k}\left(m,n\right)-I_{k}\left(m+u,n+v\right)\\ =\eta_{k}\left(m,n\right)-\eta_{k}\left(m+u,n+v\right)\sim N\left(0,2\sigma^{2}\left(f\right)\right) \end{array}$$

The pixel level of a noise-free image will change after being affected by noise. Assuming that the flat area with pixel level *f* is affected by the overall noise standard deviation σ(f), there is no texture intensity jump between $I_{k}\left(m,n\right)$ and its neighboring pixel $I_{k}(m+u,n+v)$, we denote this probability as $P_{0}$. Otherwise, the probability is recorded as $P_{1}$. Then, the following deduction can be then acquired:

(14) $$\begin{array}{l} P_{0}=P\left\{L_{d}=0\right\}=P\left\{\left|I_{k}\left(m,n\right)-I_{k}\left(m+u,n+v\right)\right|\leq \delta \right\}\\ =\frac{1}{\sqrt{2}\sigma \left(f\right)\sqrt{2\pi }}\int_{-\delta }^{\delta }e^{-\frac{x^{2}}{2{\left(\sqrt{2}\sigma \left(f\right)\right)}^{2}}}dx=\frac{1}{\sigma \left(f\right)\sqrt{\pi }}\int_{0}^{\delta }e^{-\frac{x^{2}}{4\sigma^{2}\left(f\right)}}dx\\ \underline{\underline{x=2\sigma \left(f\right)t}}\frac{2}{\sqrt{\pi }}\int_{0}^{\frac{\delta }{2\sigma \left(f\right)}}e^{-t^{2}}dt=erf\left(\frac{\delta }{2\sigma \left(f\right)}\right),d\in \left[0,7\right] \end{array}$$

where erf(•) is the Gauss error function. Therefore, $P_{1}=P\left\{L_{d}=1\right\}=1-P_{0}=1-erf\left(\frac{\delta }{2\sigma \left(f\right)}\right)$ and $L_{d},d\in \left[0,7\right]$ are independent identically distributed variables and follow the 0–1 distribution. 

From Figure 1, we can infer that a cyclic jump occurs when the sum of any adjacent two-bits in an eight-bit binary sequence equals 1 and the corresponding probability, $P_{L}$, is computed as:

(15) $$P_{L}=P\left\{L_{7}+L_{0}=1\right\}=P\left\{L_{0}+L_{1}=1\right\}=\cdots =P\left\{L_{6}+L_{7}=1\right\}=2P_{0}P_{1}$$

Conversely, a cyclic jump is skipped when this sum does not equal 1 and the corresponding probability is, then,$1-2P_{0}P_{1}$. Therefore, $t_{k}\left(m,n\right)$ follows a binomial distribution i.e., $t_{k}\left(m,n\right)\sim B\left(8,P_{L}\right)$, and, resultantly, $T_{k}\left(m,n\right)\times {\left(N-2\right)}^{2}\sim B\left(8{\left(N-2\right)}^{2},P_{L}\right)$ is obtained.

τ is the threshold of the weakly textured image blocks and can be expressed as a function of the given noise level, as shown in (16), where ζ is the confidence level, set to 1–10^−6^ as in [18]. F^−1^ represents an inverse binomial cumulative function. The image blocks where the texture strengths are less than τ, are defined as weakly textured image blocks.

(16) $$\tau =F^{-1}\left(\zeta ,8{\left(N-2\right)}^{2},P_{L}\left(\sigma \left(f\right)\right)\right)$$

④: Estimating Pixel Intensity and Variance Sample Pairs

After selecting the weak texture image block, we need to estimate pixel intensity and variance sample pairs to prepare for the subsequent fitting of the sample pair to effective noise parameters.

Estimated pixel values of the weakly textured image blocks are expressed as an average of all pixel values within the blocks, given by ${\hat{x}}_{wk}$ for wk∈[1,B_weak_n]. B_weak_n is the number of weakly textured image blocks:

(17) $${\hat{x}}_{wk}=\frac{1}{N^{2}}\sum_{m=1}^{N}\sum_{n=1}^{N}I_{wk}(m,n),wk=1\sim B\_weak\_n$$

where wk is the index of weakly textured image blocks, B_weak_n is the total number of weakly textured image blocks.

In this study, the characteristics of natural images are used to estimate the noise level. Due to the redundancy of natural images, the data of natural images only span low-dimensional subspaces, so the noise variance of weak texture image blocks can be estimated based on principal component analysis (PCA), thereby producing better estimation results.

Consider the variance of the data projected to a certain direction u, the definition of the minimum variance direction $u_{min}$ is as follows:

(18) $$u_{min}=arg\underset{u}{min}Var(u^{T}I_{wk})$$

The noise variance ${\hat{\sigma }}_{wk}^{2}$ of a weakly textured image block $I_{wm}$ is computed as:

(19) $${\hat{\sigma }}_{wk}^{2}={\left\|u_{\min}^{T}I_{wm}\right\|}^{2},m\in \left[1,B\_weak\_n\right]$$

where $\left\|u_{\min}^{T}I_{wm}\right\|$ is the Euclidean norm of the vector $u_{\min}^{T}I_{wm}$, and $u_{\min}$ is the minimum variance direction vector calculated using PCA and defined as the eigenvector associated with the minimum eigenvalue of the covariance matrix given in (20).

(20) $$C_{P}=\frac{1}{B\_n}\sum_{k=1}^{B\_n}I_{k}I_{k}^{T}$$

where *Cp* is the covariance matrix. Thus, the sample pairs of pixel intensity and noise variance estimated from the weakly textured image blocks is $\left({\hat{x}}_{wk},{\hat{\sigma }}_{wk}^{2}\right),wk\in \left[1,B\_weak\_n\right]$.

⑤: Fitting Sample Pairs to Elicit Noise Parameters 

The ordinary least squares method was used to fit the sample pairs and obtain the measurable parameters $\hat{a}$ and $\hat{b}$. Here, we define three parameters viz.$X={\left[{\hat{x}}_{1},{\hat{x}}_{2},L,{\hat{x}}_{m},L,{\hat{x}}_{B\_weak\_n}\right]}^{T}$, $X_{1}=[X,1]$, a matrix of size B_weak_n×2, where the first column vector is *X* and the second is 1, and $Q={\left[{\hat{\sigma }}_{1}^{2},{\hat{\sigma }}_{1}^{2},\cdots ,{\hat{\sigma }}_{m}^{2},\cdots ,{\hat{\sigma }}_{B\_weak\_n}^{2}\right]}^{T}$. The measurable parameters $\hat{a}$ and $\hat{b}$ are then fitted using a least squares method given as:

(21) $${\left[\hat{a},\hat{b}\right]}^{T}=\underset{{\left[a,b\right]}^{T}}{argmin}{\left\|X_{1}{\left[a,b\right]}^{T}-Q\right\|}^{2}$$

Modules ③–⑤ are conducted iteratively to acquire optimal noise parameters as in [18]: (a) An initial noise variance is estimated using all blocks in the supplied noisy image; (b) weakly textured image blocks are selected from step ③ by using the initial noise variance; (c) the current image noise variance is estimated from the weakly textured image blocks in modules ④–⑤ and (6); and (d) module ③ is revisited to initiate a new iteration of obtaining another set of weakly textured image blocks based on the current noise variance. The process is complete when the estimated noise variance remains unchanged.

## 4. Experimental Results

The experiments were performed in MATLAB 2016a on a computer with 3.30 GHz Intel Pentium G3260 CPU and 4 GB random access memory.

**Figure 2:**
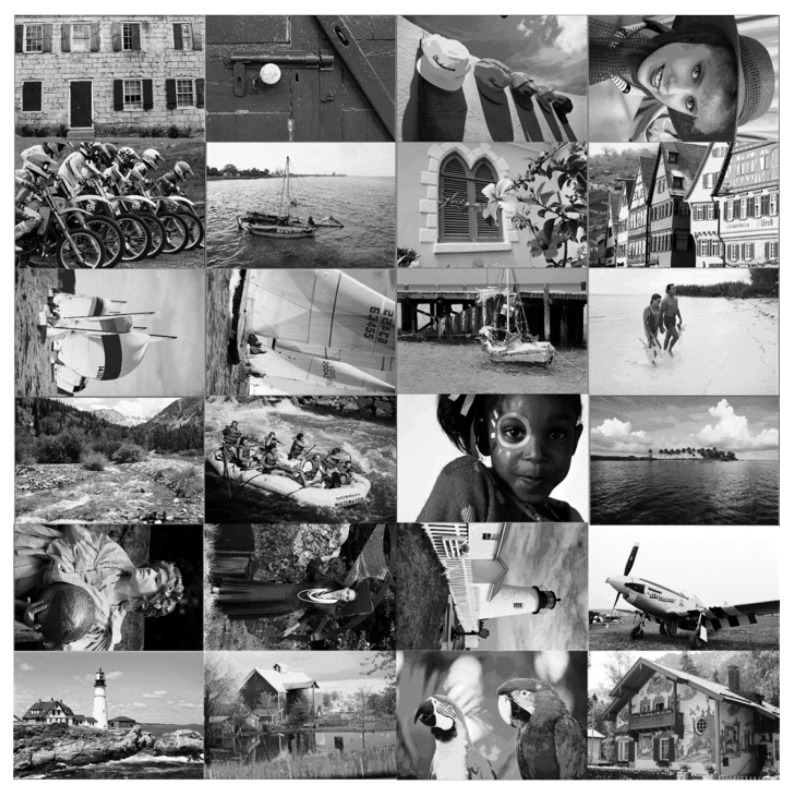
Test set: 24 noise-free Kodak PCD0992 images.

A test set of 24 true standard Kodak PCD0992 noise-free images [26], as shown in Figure 2, was adopted for the experiment. A signal-dependent noise was added to them in compliance with Equations (1)–(6). 16 pairs of noise parameters were chosen and expressed as a pairwise combination of *a* = {0.005, 0.010, 0.015, 0.020} and *b* = {0.0016, 0.0036, 0.0064, 0.0100}.

Because almost all denoising methods are based on gray image [27], we changed the three-dimensional RGB full-color image into one-dimensional gray image to process. The graying formula is as follows:

(22) Gray=0.30R+0.59G+0.11B

where *R*, *G*, and *B*, respectively, represent three channels of the RGB image.

The proposed method was validated against four existing state-of-the-art noise parameter estimation methods based on: (a) image gradient matrix [18]; (b) image histogram [19]; (c) image local gray entropy [20]; and (d) CNN [28], which has achieved superlative results. 

In order to fairly verify whether the proposed technique improved the accuracy of estimating noise parameters a and b, the operations of all steps of the algorithm, besides selecting the weakly textured image blocks, remained consistent across the board. 

The mean square error (MSE) was used to measure the accuracy of the noise estimation result. The smaller the mean square error, the better the estimation result. MSE values of the 24 test images given in Figure 2, for each parameter setting, from the following sixteen sets, i.e., (*a* = 0.005, *b* = 0.0016); (*a* = 0.005, *b* = 0.0036); (*a* = 0.005, *b* = 0.0064); (*a* = 0.005, *b* = 0.0100); (*a* = 0.01, *b* = 0.0016); (*a* = 0.01, *b* = 0.0036); (*a* = 0.01, *b* = 0.0064); (*a* = 0.01, *b* = 0.0100); (*a* = 0.015, *b* = 0.0016); (*a* = 0.015, *b* = 0.0036); (*a* = 0.015, *b* = 0.0064); (*a* = 0.015, *b* = 0.0100); (*a* = 0.020, *b* = 0.0016); (*a* = 0.020, *b* = 0.0036); (*a* = 0.020, *b* = 0.0064); (*a* = 0.020, *b* = 0.0100), were calculated as follows:

(23) $$MSE\left(a\right)=\frac{1}{M}\sum_{i\_tI=1}^{M}{\left({\hat{a}}_{i\_tI}-a\right)}^{2},MSE\left(b\right)=\frac{1}{M}\sum_{i\_tI=1}^{M}{\left({\hat{b}}_{i\_tI}-b\right)}^{2}$$

where (a,b) is a set of preconfigured noise parameter values, $\left({\hat{a}}_{i\_tI},{\hat{b}}_{i\_tI}\right)$ is the estimated noise parameter values, i_tI is the index of the test image, and M is the total number of test images (i.e., 24 for this study).

**Figure 3:**
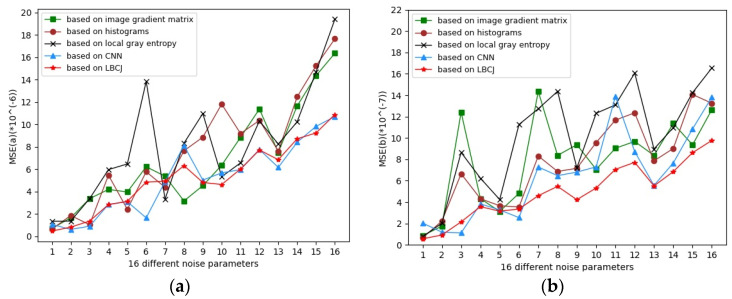
MSE comparison results of different parameter estimation methods. (**a**) Comparison results of MSE (a); (**b**) comparison results of MSE (b).

Figure 3 enumerates the comparative MSE results of the various noise parameter estimation methods cited in this study with linear least squares fitting. From Figure 3, it is evident that the proposed estimation method obtains lower and smoother MSE values compared to those of other methods, which is attributed to the conscientious selection of weakly textured image blocks using LBCJ. Considering that LBCJ captures the change in the pixel values in the eight-neighbor connected domain of the central pixel in multiple directions, it interprets the textural alterations, and thereby selects weakly textured image blocks more accurately, which in turn warrants a more precise parameter estimation.

**Figure 4:**
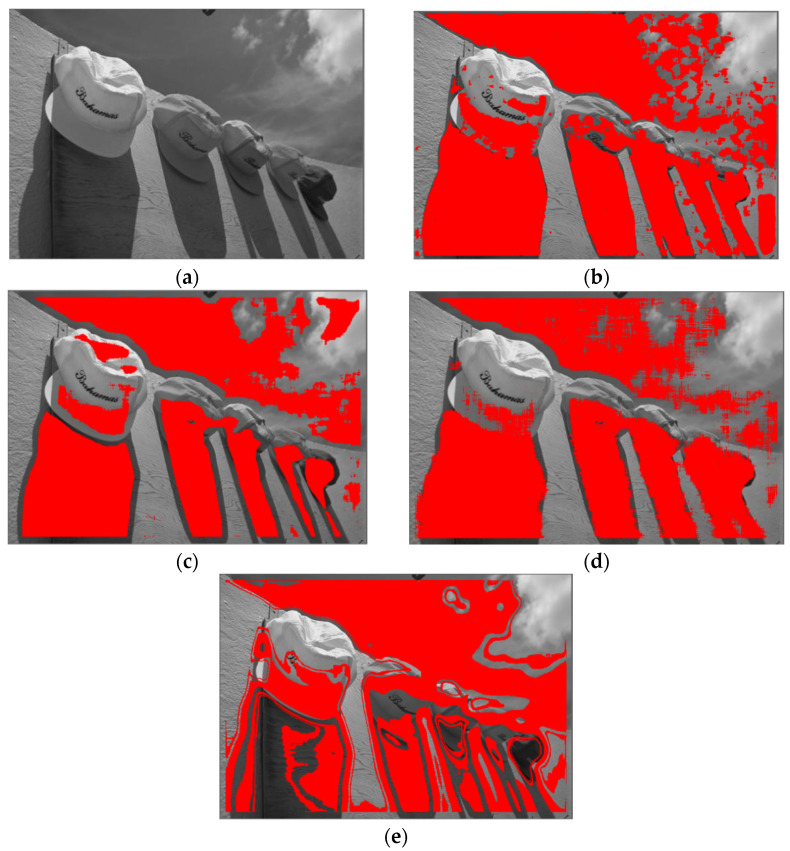
Weakly textured blocks selected by different methods. (**a**) Original image; (**b**) selection results based on LBCJ; (**c**) selection results based on grey entropy; (**d**) selection results based on gradient matrix; and (**e**) selection results based on histogram.

Figure 4 shows the visual comparison of weakly textured image block selection results under different methods. The proposed method was compared with the existing methods based on local grey entropy, image histogram and image gradient matrix, which are all the methods based on the selection of weakly textured image block. The noise parameter was set to *a* = 0.005, *b* = 0.0016. It can be seen from Figure 4 that each method can obtain a good selection result of weakly textured image blocks. In areas where the texture changes frequently, such as the cloud in the upper right corner, the proposed method can select the weakly textured image blocks more accurately.

## 5. Computational Complexity

The running time and memory consumption of the algorithm we proposed is related to the size of N. In order to compare the running time and memory consumption of our algorithm and other methods, we further tested our method in MATLAB 2016a on a computer with 3.30 GHz Intel Pentium G3260 CPU and 4 GB random access memory. For fair comparison, the competing methods were also tested in the same environment. We selected one of the Kodak pictures and added 16 sets of noise to it respectively, the results are compiled in Table 1 and Table 2. With the comparison of running time, as can be seen, our method was faster than the other three competing methods. In addition, when the noise intensity increased significantly, the running time of the other three methods increased significantly, while the running time of our method was relatively kept within a relatively small change interval. With the comparison of memory consumption, we can see that the memory consumption of our proposed algorithm was smaller than that of method based on image gradient matrix and method based on local grey entropy, which was similar to that of method based on image histogram. In general, our algorithm has faster running speed and smaller memory consumption.

## 6. Conclusions

This study proposed a new methodology of determining the noise parameters of a Poisson–Gaussian signal-dependent noise using local binary cyclic jumping. Through calculating the LBCJ information in the eight neighborhoods around the pixel to define the texture intensity and using the binomial cumulative function to determine the selection threshold, the weakly textured image blocks can be selected more accurately. The experimental results have shown that the proposed algorithm solicits lower MSE values and exhibits a superior performance over those of the existing algorithms. Therefore, it can be considered that the proposed algorithm can help improve the image quality of the CMOS image sensors and other digital imaging systems.

## Figures and Tables

**Table 1:** Running time comparison.

Noise Parameters		Time (s)			
*a*	*b*	Image Gradient Matrix	Local Grey Entropy	Image Histogram	LBCJ
0.005	0.0016	12.72	19.56	19.22	11.66
0.005	0.0036	12.86	19.59	18.56	11.45
0.005	0.0064	12.71	19.55	18.67	11.51
0.005	0.0100	12.69	19.55	18.56	11.25
0.010	0.0016	15.87	19.45	18.89	11.40
0.010	0.0036	15.53	19.56	18.52	11.39
0.010	0.0064	16.48	19.66	18.52	12.17
0.010	0.0100	16.14	19.59	18.55	12.46
0.015	0.0016	15.54	19.68	18.64	13.17
0.015	0.0036	15.97	19.52	19.03	13.51
0.015	0.0064	15.61	19.56	18.88	11.87
0.015	0.0100	15.33	19.57	19.04	12.01
0.020	0.0016	22.52	30.56	20.52	12.36
0.020	0.0036	22.18	30.59	20.52	12.06
0.020	0.0064	22.45	30.52	20.62	12.68
0.020	0.0100	21.60	30.61	20.83	12.33

**Table 2:** Memory consumption comparison.

Noise Parameters		Memory Consumption (MB)			
*a*	*b*	Image Gradient Matrix	Local Grey Entropy	Image Histogram	LBCJ
0.005	0.0016	3738	3721	3507	3513
0.005	0.0036	3741	3719	3500	3518
0.005	0.0064	3799	3716	3515	3511
0.005	0.0100	3797	3715	3512	3500
0.010	0.0016	3775	3730	3500	3499
0.010	0.0036	3770	3722	3488	3512
0.010	0.0064	3749	3729	3487	3510
0.010	0.0100	3744	3743	3525	3517
0.015	0.0016	3775	3728	3446	3340
0.015	0.0036	3769	3727	3453	3354
0.015	0.0064	3762	3721	3452	3428
0.015	0.0100	3753	3720	3463	3462
0.020	0.0016	3733	3731	3472	3469
0.020	0.0036	3733	3733	3470	3463
0.020	0.0064	3731	3731	3469	3464
0.020	0.0100	3742	3732	3452	3500

## Data Availability

The Kodak test dataset used in the experiment comes from http://r0k.us/graphics/kodak/ (accessed on 1 March 2018).

## References

[1] Li Z.P., Jiang M., Zhang X.N., Chen X.Y., Hou W.K. (2017). Space-time-multiplexed multi-image visible light positioning system exploiting pseudo-miller-coding for smart phones. IEEE Trans. Wirel. Commun..

[2] Cao C., Shirakawa Y., Tan L., Seo M.W. (2019). A time-resolved NIR lock-in pixel CMOS image sensor with background cancelling capability for remote heart rate detection. IEEE J. Solid-State Circ..

[3] Hasan A.M., Melli A., Wahid K.A. (2018). Denoising low-dose CT images using multiframe blind source separation and block matching filter. IEEE Trans. Radiat. Plasma Med. Sci..

[4] Ma X.L., Hu S.H., Yang D.S. (2019). SAR Image De-noising Based on Residual Image Fusion and Sparse Representation. KSII Trans. Internet Inf. Syst..

[5] Xu J.T., Nie H.F., Nie K.M., Jin W.M. (2017). Fixed-pattern noise correction method based on improved moment matching for a TDI CMOS image sensor. J. Opt. Soc. Am. A.

[6] Han L.Q., Xu J.T. (2018). Long exposure time noise in pinned photodiode CMOS image sensors. IEEE Electr. Device Lett..

[7] Ding L., Zhang H.Y., Xiao J.S., Lei J.F., Xu F., Lu S.J. (2020). Mixed Noise Parameter Estimation Based on Variance Stable Transform. CMES-Comput. Model. Eng. Sci..

[8] Ehret T., Davy A., Morel J.M. (2019). Model-blind video denoising via frame-to-frame training. Comput. Vis. Pattern Recognit..

[9] Yi W., Qiang C.Q., Yan Y. (2010). Robust impulse noise variance estimation based on image histogram. IEEE Signal. Proc. Lett..

[10] Foi A., Trimeche M., Katkovnik V., Egiazarian K. (2008). Practical Poissonian–Gaussian noise modeling and fitting for single-image raw-data. IEEE Trans. Image Process..

[11] Pham T.D. (2015). Estimating parameters of optimal average and adaptive wiener filters for image restoration with sequential Gaussian simulation. IEEE Signal. Proc. Lett..

[12] Pyatykh S., Hesser J. (2014). Image sensor noise parameter estimation by variance stabilization and normality assessment. IEEE Trans. Image Process..

[13] Mäkitalo M., Foi A. (2014). Noise parameter mismatch in variance stabilization with an application to Poisson–Gaussian noise estimation. IEEE Trans. Image Process..

[14] Huang X.T., Chen L., Tian J., Zhang X.L. (2015). Blind image noise level estimation using texture-based eigenvalue analysis. Multimed. Tools Appl..

[15] Jeong B.G., Kim B.C., Moon Y.H., Eom I.K. (2014). Simplified noise model parameter estimation for signal-dependent noise. Signal. Process..

[16] Zhang Y., Wang G., Xu J. (2018). Parameter estimation of signal-dependent random noise in CMOS/CCD image sensor based on numerical characteristic of mixed Poisson noise samples. Sensors.

[17] Zhang Y., Wang G., Xu J. (2014). The modified gradient edge detection method for the color filter array image of the CMOS image sensor. Opt. Laser Technol..

[18] Liu X., Tanaka M., Okutomi M. (2014). Practical signal-dependent noise parameter estimation from a single noisy image. IEEE Trans. Image Process..

[19] Dong L., Zhou J., Tang Y.Y. (2018). Effective and fast estimation for image sensor noise via constrained weighted least squares. IEEE Trans. Image Process..

[20] Li Y., Li Z., Wei K. (2019). Noise estimation for image sensor based on local entropy and median absolute deviation. Sensors.

[21] Chen J., Chen J., Chao H. (2018). Image blind denoising with generative adversarial network based noise modelling. Comput. Vis. Pattern Recognit..

[22] Guo S., Yan Z., Zhang K. (2019). Toward convolutional blind denoising of real photographs. Comput. Vis. Pattern Recognit..

[23] Zhu S., Xu G., Cheng Y. (2019). BDGAN: Image blind denoising using generative adversarial networks. Pattern Recognit. Comput. Vis..

[24] Tan Z., Li K., Wang Y. (2021). Differential evolution with adaptive mutation strategy based on fitness landscape analysis. Inf. Sci..

[25] Tan Z., Li K. (2021). Differential evolution with mixed mutation strategy based on deep reinforcement learning. Appl. Soft Comput..

[26] Standard Kodak PCD0992 Test Images. http://r0k.us/graphics/kodak/.

[27] Xu J., Zhang L., Zhang D. (2017). Multi-channel Weighted Nuclear Norm Minimization for Real Color Image Denoising. IEEE Comput. Soc..

[28] Mafi M. (2020). Deep convolutional neural network for mixed random impulse and Gaussian noise reduction in digital images. IET Image Process..

